# Predicting Health with Function ‐  How Can Biomechanics “Ride the Tiger”?

**DOI:** 10.1002/jcsm.12576

**Published:** 2020-07-16

**Authors:** Tobias Winkler, Georg N. Duda

**Affiliations:** ^1^ Julius Wolff Institute, Center for Musculoskeletal Surgery, Berlin Institute of Health Center for Regenerative Therapies Charité ‐ Universitaetsmedizin Berlin Berlin Germany

Gait speed is a well‐established surrogate endpoint to many clinical studies and has, specifically in geriatric evaluations, been shown to correlate with various medical compromised conditions and even patient survival.^1^, ^2^ In a recent publication, Piau et al. could identify a decline in walking speed 3 months prior to actually suffering from a fall event showing the high relevance of walking speed as indicator for functional impairment or even impending injuries.^3^ In the elderly, each 10th fall results in a major trauma such as a fracture.^4^ Such observations illustrate the significance of changes in gait speed as indicator for progression of neuro‐musculoskeletal degeneration. While gait speed is an indicator, the underlying biomechanical mechanisms that relate such speed to neuro‐musculoskeletal degeneration remain elusive and how gait speed indirectly or eventually directly affects overall patient conditions and survival remains unknown.

In their article ‘Changes in knee extension peak torque and body composition and their relationship with change in gait speed’, Yusuke Osawa *et al*. nicely evaluated the correlation of a composite measure, namely, quadriceps femoris strength against anatomical parameters such as muscle and fat mass with walking speed. They used a well‐established cohort, the Baltimore Longitudinal Study of Aging, that included 575 men and 539 women. Previous studies have mostly predicted changes in mobility based on only cross‐sectional analyses of functional or morphological measurements, which makes this study particularly valuable. In their paper, Osawa et al. described that a decline in strength powerfully correlates with a reduction in gait speed in men and women. A combinatory analysis of the change of strength and appendicular lean muscle mass (ALM) and fat mass, however, showed different results for both sexes. In men, a decline of all parameters was associated with a decline in gait speed, whereas in women, only a decline in strength and ALM and an increase in fat mass were associated with a gait speed decline.

This work raises several points of special interest: (i) the findings demonstrate that static data and data taken from one time point were not able to predict changes in gait speed. (ii) Single measurements of body composition without functional assessment did not correlate with gait speed but have been shown to influence speed if analysed together with muscle strength. (iii) This confirms that the analysis of parameters horizontal to gait speed such as the other functional parameter strength is more reliable in predictive models than the analysis of vertically oriented upstream parameters such as the structural parameters ALM or fat. If structural surrogate parameters for muscle function are analysed, however, they should be as closely as possible linked contractile muscle substance in order to be able to correlate them with muscle function. Total muscle volume without accounting for intramuscular fat has been shown to be imprecise when correlating with functional parameters.^5^, ^6^

A major challenge is that although a bouquet of analyses is existing—might they be biomechanical (strength tests) or structural (DEXA, MRI, and CT measurements) analyses or patient‐related outcome measures (PROM, disease specific, or general scores)—too few validated correlations between the single tests exist. Apparently, the identification of relevant study endpoints is challenging, and we are confronted with a lack of correlations with easily available tests of biomechanical functional and anatomical structural parameters.

Thus, we hope to see in the close future an increasing evidence that links clinically meaningful outcome parameters with functional or anatomical surrogates. Eventually, combinations of surrogates—as discussed by Osawa *et al*.—might help.

Looking at the clinical relevance of the analysis of anatomical morphological and biomechanical functional parameters for muscles, one of the major determinants is whether or not they are accessible for therapeutic interventions. While neuro‐musculoskeletal functional impairments act at multiple levels, an ideal therapeutic strategy would act also at various hierarchical levels within a functional chain. Novel cell therapeutics for example have been shown to improve structural parameters, such as muscle volume, and functional parameters, such as muscle strength.^7^ Study data also show that therapeutics leading to a reduction of intramuscular fat can also achieve an increase in muscle strength.^8^, ^9^

In the present paper, isometric muscle strength changes are correlated with changes in gait speed and various other clinical outcomes. The knee extensors are focus of the work, mainly, because they are (i) easily accessible even in the elderly, (ii) the largest muscle group eventually representative for the overall muscle status in the human body, (iii) structural analyses of the quadriceps can be employed as an upstream parameter correlated to overall resulting strength, and (iv) knee extension strength is central to stability in human gait and its status is eventually affecting stumbling or fall events.^10^, ^11^ The knee flexors interact closely with other muscle groups that critically affect human gait and stability such as the gluteal muscle groups that cross the hip or—via the tractus tensor fascia latae also the knee joint. Also, the spinal erectors and many pelvic and lower spine muscle groups affect the hip and knee joint and thereby contribute to functional gait stability and are highly active in a stumbling event.^12^, ^13^ We and others could show that beside the role of single muscle groups, their co‐contraction capability is key for joint contact forces in daily living and essential for the resulting forces acting within the musculoskeletal system.^12^ Modern 3D gait assessment allows to generate, when combined with 3D MRI data, human patient siblings to characterize the neuro‐musculoskeletal functionality of the whole lower limb chain and to identify movement patterns that are triggering functional degeneration cascades and/or lead to extreme events for the patients, such as falls. These 3D gait analyses might enable scientists and clinicians to find and describe movement patterns rather than single parameters such as muscle strength or gait speed to be ideal predictors of clinical outcomes.

On the other hand, there is an ongoing discussion on how to determine clinical outcomes and whether the established tools, such as PROMs, are sufficiently specific to show changes on how a patient feels, functions, or survives.^7^ The here cited Harris Hip Score, a score initially developed for patients being treated with an archaic method, mould arthroplasties in posttraumatic arthritis,^14^ but widely used for many other diagnoses, is a very good example for this fact and a correlation also with proven and sensitive parameters such as gait speed will fail,^15^ underlining the need for the search for more specific PROM.

Sex‐specific differences are increasingly more analysed not only because of gender aspects discussed in social contexts but also because we have increased our scientific insight about sex‐related differences in various fields. Against the background of still vastly homogenized and undifferentiated diagnostics and therapies, these data are decisive but have yet to merge with clinical routine. Despite personalized medicine being on the top agenda of public institutions such as the European Union research actions and initiatives, we have not yet managed to fully integrate our knowledge of sex differences into diagnostic and therapy algorithms. The data from Osawa *et al*., who found significant differences in the correlations between muscle structure and strength versus gait speed between female and male individuals, indicate that, when looking at skeletal musculature, this seems to be especially true and therefore must be considered.

Assessing the elderly population and preventing rather than treating functional incapability and accompanying diseases will be the great and demanding task of the future. The prediction of impending functional deterioration will be central and longitudinal analyses seem to be key for that.

## Conflict of Interest

None declared.
